# Association between sleep quality and depression among institutionalized and community older people - Brazilian Western Amazonia

**DOI:** 10.1186/s12888-021-03368-y

**Published:** 2021-07-23

**Authors:** Cleide Maria de Paula Rebouças, Maura Regina Ribeiro, Juliana Zangilorami-Raimundo, Polyana Caroline de Lima Bezerra, Angelo Márcio das Chagas de Souza Júnior, Nair da Silva Souza, Janaina Ribeiro Pereira, José Maria Soares Júnior, Larissa Maria de Paula Rebouças da Costa, Luiz Carlos de Abreu, Rodrigo Daminello Raimundo

**Affiliations:** 1 Laboratório de Delineamento de Escrita Científica da Faculdade de Medicina do ABC, FMABC, Santo André, São Paulo, Brazil; 2 Laboratório Multidiciplinar de Estudos e Escrita Científica em Ciências da Saúde – LAMEECCS, Universidade Federal do Acre- Brazil, Rio Branco, Brazil; 3 Laboratório de Delineamento de Estudos e Escrita Científica-LABDEEC da UNINORTE, Rio Branco, Acre Brazil; 4 Department of Acre (SESACRE), Rio Branco, Acre Brazil; 5 Preceptor of Psychogerontology Medicine Course at the University Center UNINORTE, Rio Branco, Acre Brazil; 6 Disciplina de Ginecologia, Departamento de Obstetrícia e Ginecologia, Faculdade de Medicina FMUSP, Universidade de São Paulo, São Paulo, SP Brazil

**Keywords:** Older, Depression, Sleep disorder, Institutionalized older health, Primary health care

## Abstract

**Background:**

The transition in the population pyramid is a reality in several locations around the world and projections of an increase in the older population in Brazil demonstrate the relevance of studies on factors that may interfere in the functionality and quality of life in this age group. Thus, the present study aims to assess depression levels and their relationship with sleep quality in institutionalized and community older adults.

**Methods:**

This cross-sectional study included 220 older people of both sexes, divided into two groups, institutionalized older adults, and community older adults. The older adults were monitored by Community Health Agents (CHA), through identification of everyone in their micro area using a method of random name generation, based on geographic location. Due to the small number of institutionalized older adults, all residents in the institutions were recruited, according to the inclusion and exclusion criteria. The Geriatric Depression Scale (GDS-15) and Pittsburgh Sleep Quality Index (PSQI) were used to assess depression and sleep quality.

**Results:**

Among the 220 older adults, 175 were community members and 45 were institutionalized. The survey revealed that institutionalized older adults had a higher percentage of severe depression compared to community dwelling older adults (*p* <  0.039).

**Conclusion:**

Older adults in the community present greater symptoms of depression and better sleep than institutionalized older adults. There was a direct association between sleep quality and depression. In our sample, being institutionalized and female positively influenced and feeling alone negatively influenced depressive symptoms.

**Supplementary Information:**

The online version contains supplementary material available at 10.1186/s12888-021-03368-y.

## Introduction

There is a global projection of approximately two billion older adults for the year 2050, 21% of the world population. In this respective period, 30% of the internal population of 64 countries will be over the age of 65 years [1, 2].

In 2010, 8.6% of the population of Brazil was composed of older adults, with a projection of 13% for 2020. For 2050, the projection is that the number of older adults will exceed that of people under 15 years of age, corresponding to 20% of the Brazilian population [3].

Population aging in both developed and developing countries has caused economic impacts on health and social security systems worldwide, due to the lack of adequate preparation in these nations [4].

Parallel to population aging, there will be an increase in chronic and psychological diseases (sleep problems, psychological distress, obesity, diabetes, and metabolic syndrome) with variable impacts on physical, cognitive, and mental function [5].

Fang et al. (2019) [6] identified that sleep disorders affect a quarter of the world’s population and can predispose to mental disorders, especially depression. Sleep is a part of the body’s daily cycle. Observational and experimental studies have shown that short sleep duration and sleep disorders are associated with poor performance in daily tasks, depressive disorders, memory problems and reduced academic performance [7–9], reduced motivation, suicidal thoughts, and obesity [8].

Sleep problems prevail in poor psycho-physiological health conditions, such as stress, anxiety, fatigue, depression, attention deficit, reduced cognitive performance, and impaired social relationships [9, 10]. Insomnia can occur before the onset of a mental disorder, simultaneously, or can even appear later. In the Diagnostic and Statistical Manual of Mental Disorders (DSM-V), insomnia was identified as a primary diagnosis or as a comorbidity with other mental and medical disorders [5] which interferes with functional capacity, and that can be considered a primary disorder or associated with other medical and psychiatric disorders [11].

The association of the influence of the epidemiological and sociodemographic profile with levels of depression and its relationship with self-reported sleep quality in older adults is indicative of the importance of establishing Public Policies focusing on improving the quality of life of the older community [12].

Due to geographical conditions, population distribution, and precarious living conditions, some health situations affect the population of the Amazon region differently from other regions of Brazil, such as: low income, painful working conditions, high occupational risks, violence, intense and recurrent exposure to infectious agents, lack of access to quality housing and sanitation, and limited access to health care and care actions in primary care in the region [13]. Therefore, research in Primary Health Care, as well as with institutionalized older adults in a region where there is little effectiveness of public health policies, could contribute to the identification of health determinants in the Amazon region.

In summary, the present study aims to assess levels of depression and the relationship with sleep quality in institutionalized and community older adults. We hypothesized that there is an association between bad sleep quality and depression, and that this is more intense in institutionalized seniors.

## Methods

### Sample

A cross-sectional study was conducted during February 2017 to March 2019, in the State of Acre, Brazil, located in the extreme southwest of the Northern region, in the middle of the Brazilian Amazon in the cities of Rio Branco, Taraucá, and Cruzeiro do Sul. The total population of the state of Acre was 733,559 in the December 2010 Census. The older population of Acre was 26,841 (corresponding to 6.38% of the population) [13].

The scope of Primary Health Care (PHC) in Rio Branco in 2019 corresponded to 95.5%. The research was carried out in four Basic Health Units (BHU) in Rio Branco, and in four Long-Stay Institutions in different municipalities in the state of Acre. There are only six health units in the municipality of Rio Branco and the study was carried out in four of them.

The study population of older people followed by the Family Health Strategy (FHS) teams corresponded to 2138 individuals. After the sample calculation, 426 older adults were selected in total. The inclusion criteria for all participants were: living in the area covered by the Health Unit investigated for at least 3 years and agreeing to participate in the research freely by signing the Consent Form (CF).

The exclusion criteria for participation in the study were having a diagnosis of organic pathologies (hearing and vision problems), psychiatric diseases, and neurological diseases (Alzheimer’s and other dementias), with impairment in relation to the transmission of thought/feeling that could make it impossible to apply the protocol instruments. Losses were considered in cases where the subjects refused to answer the questionnaire and those who were not found, after two unsuccessful attempts, at the place of residence.

**Fig. 1:**
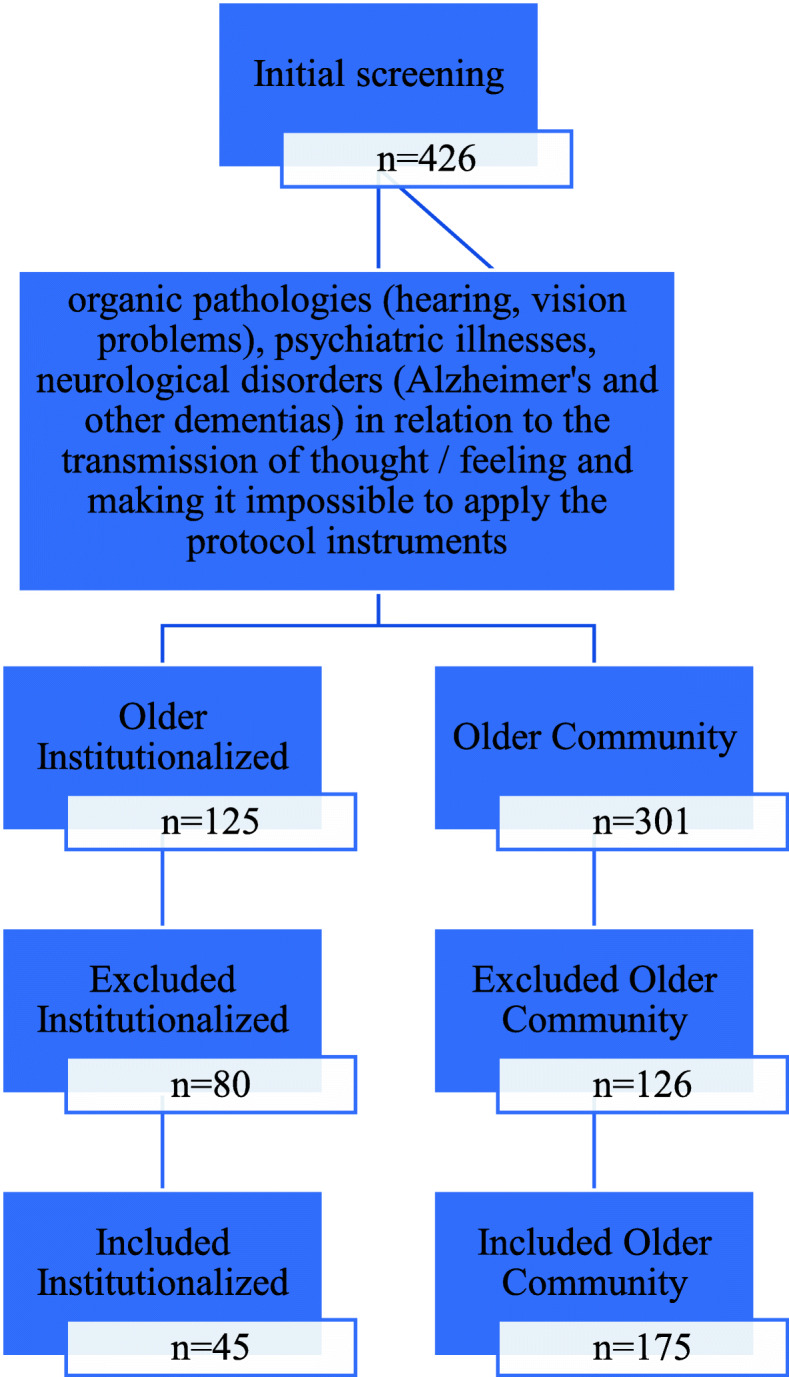
Flowchart of the studied population

After applying the exclusion criteria, the sample consisted of 175 older community members (community group), of both sexes, accompanied by Community Health Agents (CHA) from the Family Health Strategy (FHS) teams, and a second group composed of 45 older adults (institutionalized group), of both sexes, residing in Long-stay institutions (LSI), in Rio Branco (Fig. 1).

The evaluations were performed by four researchers throughout the protocol. The researchers were trained regarding the procedures to be adopted, considering the objectives of the study.

A survey of all older people who are followed by Primary Care in the city of Rio Branco was carried out by the Primary Care Information System (PCIS), and the basic health units (BHU) that had the highest number of registered older adults were chosen. We surveyed all the older adults accompanied by the CHA, who provided a list of the older adults in their micro areas. The random generation of names method was used, and the inclusion/exclusion criteria of the research were applied, with subsequent data collection based on geographic location, performed through home visits and through the CHA monitoring from the respective BHU.

In the Long-stay institutions (LSI), a list of all the older adults in the institution was provided, followed by application of the inclusion and exclusion criteria by the person responsible for the institution, who then provided the list of older adults who corresponded to the profile of the research. The protocol was applied after completion of the Consent Form (CF).

### Community older adults

A survey of all older people followed by Primary Health Care (PHC) in the city of Rio Branco was carried out, and the BHUs with the highest number of registered older adults were chosen. The choice of health units considered the criterion of having the highest number of registered older adults in the health information system - Primary Care Information System (PCIS). In these places there is a multidisciplinary Family Health Strategy (FHS) team that is the main route of entry into the Unified Health System (UHS).

We surveyed all the older adults accompanied by the CHA, who provided a list of the older adults followed in their micro areas. The random generation of names method was used, and the inclusion/exclusion criteria of the research were applied, with subsequent data collection based on geographic location, performed through home visits and through the CHA monitoring of the respective BHU.

### Institutionalized older adults

LTIs are governmental or non-governmental institutions that serve as homes for older adults, with or without family support, with the necessary maintenance of freedom, dignity, and citizenship [14].

A survey was conducted with the Secretary of State for Health of Acre to gather information about existing Long-stay institutions (LSI) and shelters. Prior contact was established with the coordinators of these institutions, who provided a list of all older residents.

In the Long-stay institutions, the coordinators of the institution were presented with the inclusion and exclusion criteria of the institution, and they made available the list that corresponded to the profile of the research and then the Consent Form was presented to the older adults, and the research protocol was applied. Data collection of older residents in the Long-stay institutions was performed at the institution premises.

The study received approval from the Ethics and Research Committee of Uninorte, under protocol number 2495347. The investigation was in accordance with the principle of volunteering and permission was received in writing. Data collection started only after authorization from the LTI coordinators and the Municipal Health Secretariat of Rio Branco, Acre, Brazil, upon signing the Consent Form (CF) by the investigated older adult or their guardian.

#### Questionnaire

A survey was carried out of all older adults monitored by Primary Health Care (PHC), considering the criterion of having the largest number of registered older adults obtained in the health information system. Then, contact and scheduling of interviews was made with the coordinators of the UBS. The PHC team made an initial approach to potential participants that they considered capable of understanding the study and passed on all the details to the older adult. The evaluations were carried out by two researchers. The researchers were trained in the procedure to be adopted considering: the objective of the research, the procedure for applying the questionnaires, and the appropriate form of communication with the establishment and with the older adults and their families. The researchers read each question out loud and recorded the responses of the older adult to the sociodemographic questionnaire and the instruments used. Each interview lasted approximately 30–45 min and was divided into three stages.

In the first stage, the sociodemographic indicators (supplementary file) were evaluated, and the following variables were collected: age, sex, marital status, education, skin color, family conflicts, loneliness, religious activity, shopping, sewing, embroidery, knitting, smoking, drinking, comorbidities (hypertension, diabetes, hypertension/ diabetes). Family conflicts corresponded to situations of incomprehension regarding the needs of the older adult due to differences of opinion with family members of different generations. Loneliness corresponded to a distressing feeling accompanied by an intuitive perception of an unpleasant, painful experience, and a feeling of non-belonging, where the person feels alone. Religious Activity corresponded to the perception of believing and practicing a religion, involving organizational (public participation), and non-organizational activities (activities outside a religious institution).

In the second stage, data on depression and self-reported sleep quality were collected using the following instruments: Geriatric Depression Scale (GDS-15) and Pittsburgh Sleep Quality Index (PSQI).

The assessment of the presence or absence of depressive symptoms was performed using the GDS-15, a reduced version of the original scale, prepared by Sheikh & Yesavage (1986) and validated in Brazil [15] This scale is used to assess symptoms of depression in older patients, consisting of 15 questions. In the GDS-15, questions are answered as “yes” or “no.” A score below five points is considered as no symptoms suggestive of depression and a total score greater than 5 indicates the possibility of depression.

The final stage, the PSQI is a tool that assesses self-reported sleep quality and disturbances during the previous month, to discriminate patients between “good sleepers” and “bad sleepers”. The total sum can reach 21 points; a total PSQI score ≤ 5 indicates “good sleepers” and a score > 5 indicates “poor sleepers”. The PSQI domains are as follows: 1) Subjective sleep quality: individual perception regarding sleep quality; 2) Sleep latency: time needed to induce sleep; 3) Sleep duration: how long you stay asleep; 4) Usual sleep efficiency: relationship between the number of hours slept and the number of hours spent in bed; 5) Sleep disorders: presence of situations that compromise the hours of sleep; 6) Use of medication to sleep, and finally, 7) Daytime sleepiness and disturbances during the day, such as willingness and enthusiasm to perform routine activities [16]. To assess the sociodemographic indicators, a specific questionnaire was constructed (supplementary file). For self-reported depression and sleep quality, the following instruments were used: Geriatric Depression Scale (GDS-15) [15] and Pittsburgh Sleep Quality Index Scale (PSQI) [16], all of which are validated to be applied in Brazil, and widely used in population-based studies. However, no validated study was identified for this specific age group.

#### Statistical analysis

Excel programs were used to prepare the database and statistical analyses were performed using SPSS 22.0 software (Statistical Package for Social Research). The descriptive analysis used median, and 25th and 75th percentiles for numerical variables, and frequency for categorical variables. The numerical variables were tested using the Mann-Whitney test, and the categorical variables using the Chi-square with Bonferroni post-hoc and Fisher’s exact test. For this work, a significance level of 0.05 was defined.

Multiple linear backward regression analysis was performed to verify which factors influenced the GDS-15. This method was used to evaluate which variables accounted for the most variance in the model, removing those that did not render a good fit and providing insight into depression scale score. The variance inflation factor (VIF) (< 10) and Tolerance (> 0.1) were checked to avoid multicollinearity problems for each independent variable. The homoscedasticity (normal distribution of residuals) was checked by inspecting the standardized residual using predicted plots. Durban–Watson’s d tests were used to examine autocorrelation in the data, with d values 0–4 indicating no autocorrelation. All tests were two-tailed, and *p* <  0.05 was considered significant.

The sample size for the frequency in the population was calculated using an older population in the State of Acre, which, according to the IBGE (Brazilian Institute of Geography and Statistics) is 54,725 older people. According to a study published in the Brazilian Journal of Geriatrics and Gerontology, 46.8% of the older people in Acre have sleep disorders (Amaral et al), therefore, the value of 54,725 was used for the population size (n); 46.8% for the hypothetical size frequency (%) of the outcome factor in the population (p); with confidence limits (d) of 5% and a design effect for cluster surveys (DEFF) of 1. The sample size was calculated using the formula: *n =* [DEFF * Np (1-p)] / [(d2 / Z21-α / 2 * (N-1) + p * (1-p)]. After the calculation, a sample size of 163 participants was generated for each group, needed to test significantly at 5% with an 80% test power.

## Results

The median age found was 76 years for institutionalized older adults and 69 years for community members (*p* = 0.011). The majority of the institutionalized older adults were male (66.7%), while the percentage of male community older adults was 30.9% (*p* <  0.001). Regarding marital status, the majority of the institutionalized older adults, were single (35.6%), while this percentage for community older was (13.1%) (p <  0,001). The sociodemographic variables of the two groups are presented in Table 1.

**Table 1:** Sociodemographic characterization of the sample of institutionalized and community older adults

	Institutionalized (*n* = 45)			Community (*n* = 175)			*p*-value
	Median	Percentile 25	Percentile 75	Median	Percentile 25	Percentile 75	
Age	76	67.5	83	69	64.75	77.25	0.011^a^
Sex							
Female	33.3% (16)			69.1% (121)			0.001^c^
Male	66.7% (31)			30.9% (54)			
Marital status							
Married	6.7% (3)			29.7% (52)			< 0.001^b^
Consensual union	6.7% (3)			8.6% (16)			
Single	35.6% (17)			13.1% (24)			
Divorced or separated	28.9% (14)			8.6% (16)			
Widow/widower	22.2% (10)			39.4% (69)			
Education							
Never attended school	51.1% (24)			47.1% (86)			0.814^b^
Reads and writes own name	33.3% (16)			39.4% (69)			
1 to 4 years	11.1% (5)			6.9% (13)			
5 to 8 years	4.4% (2)			4.6% (8)			
Skin color							
White	26.7% (13)			12.6% (23)			0.153^b^
Yellow	0			2.3% (4)			
Pardo^d^	64.4% (30)			72.6% (127)			
Indigenous origin	0			1.1% (2)			
Black	8.9% (4)			11.4% (21)			

^a^Mann-Whitney test; ^b^ Chi-square test, ^c^Fisher’s Exact test ^d^ In Brazil, Pardo is an ethnicity/color category used by the Brazilian Institute of Geography and Statistics (IBGE) in Brazilian censuses. It is a Portuguese word that encompasses various shades of brown, but is usually translated as “grayish-brown”

Regarding hobbies among the groups studied, sewing, embroidery, and knitting activities had a percentage of 6.7% of the institutionalized older adults and 29.7% of the older adults living in the community (*p* = 0.001). Regarding purchasing activities, 26.7% of institutionalized and 76.0% of community workers perform this activity (*p =* 0.001). In total, 29.1% of the community older adults reported isolation problems, compared to 46.7% of the institutionalized older adults, respectively (*p* = 0.076). Regarding family problems (conflicts), these were reported by 16.6% of the older adults in the community and 20.0% of the institutionalized older adults, respectively (*p =* 0.076). Considering attending churches, in the older community group this activity was performed by 82.3%, compared to 71.1% of the institutionalized older adults, respectively. (Table 2).

**Table 2:** Sociodemographic characterization of the sample of institutionalized and community older adults

	Institutionalized (*n =* 45)	Community (*n* = 175)	*p-*value
Smoker			
Yes	24.4% (11)	16.0% (29)	0.194^c^
No	75.6% (35)	84.0% (147)	
Alcohol consumption			
Yes	8.9% (4)	9.1% (17)	1.000^c^
No	91.1% (41)	90.9% (159)	
Comorbidities			
None	22.2% (10)	26.3% (46)	0.877^b^
Diabetes	6.7% (3)	5.1% (9)	
Hypertension	53.3% (25)	49.7% (87)	
Diabetes and hypertension	15.6% (7)	18.9% (34)	
Family Conflicts			
No	80.0% (37)	83.4% (146)	0.677^b^
Yes	20.0% (9)	16.6% (30)	
Loneliness			
No	53.3% (25)	70.9% (124)	0.076^b^
Yes	46.7% (22)	29.1% (51)	
Religious activity			
No	28.9% (14)	17.7% (32)	0.099^b^
Yes	71.1% (33)	82.3% (144)	
Sew, embroider, knit			
No	93.3% (42)	70.3% (123)	0.001^c^
Yes	6.7% (3)	29.7% (52)	
Go shopping			
No	73.3% (34)	24.0% (42)	0.001^c^
Yes	26.7% (13)	76.0% (133)	

^a^Mann-Whitney test; ^b^ Chi-square test, ^c^ Fisher’s Exact test

Community older people had a higher prevalence of statistically significant (*p*-value = 0.031) symptoms of depression (93.7%) compared to the institutionalized older adults (62.2%) according to the Bonferroni test. In assessing the association between PSQI and GDS-15, the values found were *X*^2^ (1) = 5.83 with *p*-value = 0.016 (Table 3).

**Table 3:** Comparison and association of depression and sleep quality between institutionalized and community older adults

	Institutionalized (*n* = 45)	Community (*n* = 175)	*p*-value	*X*^2^ (d) *p*-value
	Frequency (n)	Frequency (n)		
GDS-15				5.83 (1) 0.016
Without	17.8% (8)	6.3% (11)	0.031^a^	
With depressive symptoms	62.2% (38)	93.7% (164)		
PSQI				
Good sleeper	28.9% (14)	45.7% (80)	0.044^b^	
Bad sleeper	71.1% (33)	54.3% (95)		

^a^Chi-square test with Bonferroni post-hoc, ^b^ Fisher’s Exact test, Geriatric Depression Scale – Short (GDS-15)

*PSQI* Pittsburgh Sleep Quality Index

Multivariable linear regression analysis was used to assess predictors of the depression scale score. We found that the best-fitting predictors for the GDS-15 score of our participants were institutionalized (*p* = 0.029), female (*p* = 0.016), loneliness (*p* <  0.001) and PSQI (*p <* 0.001). Institutionalized older adults have a depression score − 0.765 points lower than community older adults. Women (female) presented a depression score − 0.692 points lower than men. Older adults who feel alone (loneliness) have 1.403 points more than elderly adults who do not feel alone. For each point increased in PSQI score (indication poorer sleep quality), an increase of 0.240 points in depression score was identified. (Table 4).

**Table 4:** Multivariable linear regression analysis of depressive symptoms performed by points scored on the Geriatric Depression Scale (GDS-15)

Variable				
Input model	Standardized coefficient β	Unstandardized coefficient B (95% Confidence Interval)	p	*R*^2^
(Constant)		7.629 (5.192 to 10.067)	< 0.001	0.209
Institutionalized	−0.175	− 0.923 (− 1.639 to − 0.207)	0.012	
Female	−0.175	− 0.764 (− 1.364 to − 0.165)	0.013	
Age	0.013	0.003 (− 0.027 to 0.034)	0.839	
Loneliness	0.286	1.290 (0.672 to 1.908)	< 0.001	
PSQI	0.235	0.137 (0.062 to 0.212)	< 0.001	
Smoker	0.104	0.573 (−0.127 to 1.272)	0.108	
Alcohol consumption	−0.025	− 0.186 (−1.095 to 0.724)	0.688	
Comorbidity	−0.02	− 0.096 (− 0.72 to 0.527)	0.761	
Married and stable union marital status	−0.086	− 0.385 (− 0.996 to 0.226)	0.216	
Conflicts	0.004	0.023 (− 0.723 to 0.770)	0.951	
Output model				
(Constant)		7.613 (6.951 to 8.274)	< 0.001	0.190
Institutionalized	−0.145	−0.765 (−1.449 to − 0.081)	0.029	
Female	−0.159	− 0.692 (− 1.254 to − 0.129)	0.016	
Loneliness	0.311	1.403 (0.823 to 1.983)	< 0.001	
PSQI	0.24	0.140 (0.067 to 0.213)	< 0.001	

(p) *p-*value; (*R*^2^) R square; (β) Beta; *PSQI* Pittsburgh Sleep Quality Index

## Discussion

The current research showed that older adults in the community have greater symptoms of depression and better sleep than institutionalized older adults. There was a direct association between sleep quality and depression. The prevalence of severe depression in institutionalized and community older adults were 62.2 and 93.7% respectively (*p* = 0.031), which converges with the study by Cybulski (2017) [17] carried out in a Long-stay institution (LSI), which found that older adults have a higher prevalence of chronic depression interfering with their quality of health. Several studies have shown a higher prevalence of depression in institutionalized older adults compared to those who live in the community [18–20].

Several studies showed the importance of considering sleep-wake disorders as a factor that influences the onset of depressive symptoms [21–24]. A longitudinal study in Japan with older adults showed that 80.0% of patients with depression had relevant complaints regarding deterioration in both the quantity and quality of sleep [25]. Hoyos et al. [26] identified that individuals in the experimental group of older people with depression had longer sleep latency and rapid eye movement sleep latency than those in the control group.

The predominance of the male sex was identified in the sample of institutionalized older people in this study, which is a peculiarity regarding the demographic characteristics of institutionalized older people from international studies and in some regions of Brazil. This is corroborated by research from the Institute of Applied Research and Economics (IPEA) in the State of Acre (2007) where 75.5% were men, possibly associated with migration to the Amazon border regions, which was predominantly male [27].

Holt et al. [28] and Jerez-Roig Study et al. [29] found that institutionalized older people experience negative health conditions, such as restrictions in social support networks, which end up limiting the establishment of social interactions and enhancing cognitive difficulties, in addition to a sedentary lifestyle, loss of autonomy, absence of family members and visits, and insufficient financial support, among other factors. The results found in the present study showed that 6.7% of institutionalized older people and 5.1% of community older people had diabetes, while 20.0% of institutionalized older people and 17.7% of community older people had depression.

Scientific evidence has associated poor quality or sleep deprivation with negative effects on intellectual performance, mood, memory, body weight control, reduced immunity, and an increased risk of diseases such as diabetes, hypertension, obesity, dementia, and depression. According to Fang et al. [6], a quarter of the world population has sleep disorders that lead to a greater predisposition to mental disorders, such as bipolar disorder and generalized anxiety disorders, suicidal ideation, and, mainly, depression. Chronic sleep difficulties among older adults have several repercussions with personal and social impact, such as: cognitive impairment, increased falls and mortality, psychosocial impairment, difficulty in ADLs, decreased health-related quality of life, and institutionalization [30]. In the present study, the correlation between the PSQI and GDS instruments was identified, confirming the results of other studies [11, 31] on sleep disorders and depression in older adults.

A population-based study [32] of 168 older people from the community conducted in a capital city in Northeastern Brazil, identified an association between poor sleep quality and depression (*p* < 0.05), but with no relationship after multivariate adjustment (*p* > 0.05). This loss of association in the adjusted model was due to the impossibility of elaborating a general prediction model with the inclusion of groups of sociodemographic variables, and lifestyle and sleep characteristics, since the large number of variables made it impossible to build an explanatory model. in view of the sample size.

### Strengths and limitations

This study had the following limitations: first, the sample size calculation was based on information from the literature in the only study published in Brazil about depression and sleep disturbance in older people, so we used the same calculation for the different samples (community and institutionalized). The number of institutionalized older people was small, however, it is noteworthy that this number corresponded to older residents in the only four long-stay institutions in the State of Acre; the study failed to list all risk factors for sleep disorders and depression as these are multifactorial. Despite the strengths of the study, the results obtained must be interpreted with caution because of the cross-sectional design that does not allow causality to be attributed to the variables, since there is the possibility of reverse causality, which can occur when the apparent exposure is a consequence of the outcome. However, the results demonstrated the importance of monitoring subjective sleep complaints, as they may be indicative of a parallel depressive state, or even precede depression.

On the other hand, this is a pioneering study in the state of Acre and it should be noted that the main strength was the accuracy in identifying factors relevant to health in the older adults. The results made it possible to raise hypotheses for future longitudinal studies, to verify the causality of the precarious health conditions among institutionalized older people and residents in the community. The findings of the present study suggest the need to maintain functional capacity, based on work to identify possible aspects of the psychophysiological system (sleep and mood) and social influences, which can cause damage to general well-being and quality of life.

## Conclusion

Older adults in the community have greater symptoms of depression and better sleep than institutionalized older adults. There was a direct association between sleep quality and depression. In our sample, being institutionalized and female positively influenced and feeling alone negatively influenced depressive symptoms.

## Supplementary Information

**Additional file 1.**

## Data Availability

The datasets used and/or analyzed during the current study are available from the corresponding author on reasonable request.

## Abbreviations

CHA: Community Health Agents
GDS-15: Geriatric Depression Scale
PSQI: Pittsburgh Sleep Quality Index
LSI: Long-stay Institution
DSM-V: Diagnostic and Statistical Manual of Mental Disorders
FHS: Family Health Strategy
PHC: Primary Health Care
UHS: Unified Health System
PCIS: Primary Care Information System
CF: Consent Form
BHU: Basic Health Units
IPEA: Institute of Applied Research and Economics
IBGE: (Brazilian Institute of Geography and Statistics)
