# Supplementary material for: Association between sleep quality and depression among institutionalized and community older people - Brazilian Western Amazonia
Source: BMC Psychiatry. 2021 Jul 23;21:367. doi: 10.1186/s12888-021-03368-y (PMC8299579; doi:10.1186/s12888-021-03368-y)
Supplement: Supplementary file 1 — Additional file 1. [file 12888_2021_3368_MOESM1_ESM.docx]

**Sociodemographic data collection**

| **DATE: ___ / ____ / _____** | |  | | |
| --- | --- | --- | --- | --- |
|  | | | | |
| 1. **Identification:** | | | | |
| Name: | | | | |
| Identity document : | | | | |
| Full address: | | | | |
| Telephone: | | | | |
| Gender: (     ) Female      (     ) Male | | | | |
| Birthday: ___ / ___ / _____ | | Age: | | |
| Birthplace: | | Nationality: | | |
| Marital status : | | | | |
| Married ( ) | | | | |
| Stable union    (      ) | | | | |
| Single ( ) | | | | |
| a widow (a) / divorced / separated    ( ) | | | | |
| Widow    ( ) | | | | |
| At school, what was the last grade / grade you completed successfully ? | | | | |
| I never went to school    (      ) | | | | |
| Reads and writes his/her own name    (      ) | | | | |
| (  ) 0-0 ( )1-4 ( ) 5-8 ( ) 9-11 ( ) 12 years or older | | | | |
|  | | | | |
| 1. **Skin color:** | | | | |
| How would you rate your skin color ? | | | | |
| White ( ) | | Yellow ( ) | Parda ( ) | |
| Indigenous origin ( ) | | Black ( ) | | |
|  | | | | |
| 1. **Do you currently live alone ?** | | | | |
| Yes (     ) | | No (     ) | | |
|  | | | | |
| 1. **Family Member (Informal Caregiver):** | | | | |
| Address: | | | | |
| Neighborhood : | | Municipality : | | |
| ZIP CODE : | | Reference point : | | |
| Tel : | | Cel : | | |
| Telephone number of a friend or relative to contact : | | | | |
|  | | | | |
| 1. **Family Situation:** | | | | |
| Marital Status: | | | | |
| Who do you live with : | | | | |
| Do you have: | | | | |
| Spouse (     ) | | Partner (  ) | Children (     ) | |
| Grandchildren (     ) | | Brothers (     ) | Other Relatives (     ) | |
| Are you satisfied with the relationship you have with your family members ? | | | | |
| Yes (     ) | | No (     ) | | |
| Your home is: | | | | |
| Bought (     ) | | Rented (     ) | | |
|  | | | | |
| **6. Professional activities:** | | | | |
| Do you receive a pension fund ? | | | | |
| Yes (     ) | | No (     ) | | |
| Pension ? | | | | |
| Paid activity before retirement : | | | | |
| Current paid activity : | | | | |
| Any other professional activity without pay: | | | | |
| For your basic needs, what do you get: | | | | |
| Provides everything and more (     ) | | It is enough (     ) | | |
| Almost enough (     ) | | It is not enough (     ) | | |
|  | | | | |
| **7. Sociability and Leisure:** | | | | |
| Shopping (     ) | | | | |
| Going to Church (Religious service) (     ) | |  | | |
| Sew, embroider, knit ( ) | |  | | |
|  | | | | |
| **8. Risk Factors and Health Problems:** | | | | |
| Do you have regular physical activity? | | | | |
| Yes (     ) | | No (     ) | | |
| Type: | | Duration: | | |
| brake Iinstance: | | With professional guidance? | | |
| If not, why? | | | | |
| Do you have sexual activity ? | | | | |
| Yes (     ) | | No (     ) | | |
| If not, why? | | | | |
| Is having (or not having) sexual activity satisfactory for you? | | | | |
| Yes (     ) | | No (     ) | | |
| Do you currently smoke? | | | | |
| Yes (     ) | | No (     ) | | |
| If not, have you already smoked? | | | | |
| Yes (     ) | | No (     ) | | |
| Do you currently drink? | | | | |
| Yes (     ) | | No (     ) | | |
| If not, have you drank ? | | | | |
| Yes (     ) | | No (     ) | | |
| Have you fallen in the last 12 months? | | | | |
| In affirmative case: | | | | |
| Reason for the fall : | | | | |
| Consequence: | | | | |
| In your opinion, how is your health ? | | | | |
| The thymus (     ) | Good (     ) | Bad (     ) | | Terrible (     ) |
| Compared to other people your age, the Mr . (a) would you say your health is good ? | | | | |
| Best (     ) | | Same (     ) | Worse (     ) | |
| You are a carrier: | | | | |
| Diabetes (     ) | | Hypertension (     ) | | |
| Drugs used and doses | | | | |
|  | | | | |
| **9. Mental health:** | | | | |
| When you look to the future, how do you feel, what are your expectations for the future? | | | | |
| It does not mention expectation, but it also does not refer to any negative statement (     ) | | | | |
| The future is described negatively or frightening or unbearable (     ) | | | | |
| Does not know/ does not respond () | | | | |
| would like you to inform me what are the most important problems in your daily life.   1 ( YES)   2 (NO) 3 (Does not know/ does not respond ) | | | | |
| Problems | YES | NOT | | NS / NR |
| Economic problems |  |  | |  |
| Health problems |  |  | |  |
| Fear of violence |  |  | |  |
| Housing problem |  |  | |  |
| Transport problem |  |  | |  |
| Family problems (conflicts) |  |  | |  |
| Isolation problems (loneliness) |  |  | |  |
| Problems with children / grandchildren |  |  | |  |
